# The first complete mitochondrial genome of *Rohanella titteya* (Cypriniformes: Cyprinidae) and its phylogenetic analysis

**DOI:** 10.1080/23802359.2025.2519215

**Published:** 2025-06-18

**Authors:** Yu-Hui Tao, Jin-Qiang Cheng, Jin-Yang Li, Cheng-Pu Lu, Jie Chen, Wei Liu

**Affiliations:** ^a^ Zhejiang Lishui Service Platform for Technological Innovations in Traditional Chinese Medicine Industry, Lishui University, Lishui, China; ^b^ College of Ecology, Lishui University, Lishui, China; ^c^ Forestry Bureau of Jinyun County, Lishui, China; ^d^ Forestry Bureau of Lishui City, Lishui, China

**Keywords:** Fish mitogenome, Sri Lankan freshwater fish, Conservation genetics, Mitochondrial DNA

## Abstract

We report the first complete mitochondrial genome of *Rohanella titteya* (Deraniyagala 1929), revealing a 16,715 bp genome containing 37 genes (13 protein-coding genes, 22 tRNA genes, 2 rRNA genes). Phylogenetic analysis based on mitochondrial genomic data of *R. titteya* and 11 Cyprinidae species showed that it clustered most closely with the mitogenome of *Puntius eugrammus*, offering no support for the recent transfer of this species to the new genus *Rohanella*. The mitogenome presented here provides a useful resource for both conservation and future Cyprinidae taxonomy.

## Introduction

*Rohanella titteya* (Deraniyagala 1929), formerly placed in *Puntius*, is a small freshwater fish belonging to the Cyprinidae family (order Cypriniformes). It is endemic to rainforest streams in southwestern Sri Lanka. Known for its striking red coloration of males (with iridescent green highlights) and peaceful behavior, this species is popular in the global aquarium trade (Mieno and Karino 2017). *R. titteya* feeds mainly on plant debris and small invertebrates, grows to about 5 cm in length, and lives 5–7 years. During breeding, females lay 226–284 eggs per clutch (Sundarabarathy et al. 2004). However, overharvesting for the ornamental trade has severely threatened wild populations, leading to its classification as Vulnerable by the IUCN in 2019 (Palmer-Newton et al. 2019).

Recent phylogenetic studies have shown that the *Puntius* genus (historically a "catch-all" group) is not a natural evolutionary unit and requires reclassification (Sudasinghe et al. 2023). For instance, based on mitochondrial (*cytb*, *cox1*) and nuclear (*rag1*, *irbp*) gene data, *Puntius titteya* was moved under a new genus *Rohanella* due to its unique traits, such as an incomplete lateral line and absence of a post-epiphyseal cranial fontanel (Sudasinghe et al. 2023). While mitochondrial genomic data for *R. titteya* are available in GenBank (AP011448), existing assemblies remain incomplete, omitting the hypervariable D-loop region critical for evolutionary inference. This limitation hinders comprehensive analyses of population divergence and phylogenetic relationships.

This study presents the first fully annotated mitogenome of *R. titteya* to test the reclassification from *Puntius* proposed by Sudasinghe et al. (2023), which relied on partial mitochondrial sequences and morphology. By analyzing whole-mitogenome structure and evolutionary signal, we evaluated whether its genetic architecture supports placement in *Rohanella* or retains ancestral affinity with *Puntius*. The results reduce taxonomic uncertainty and supply a genomic resource for conserving this vulnerable species.

## Materials and methods

**Figure 1.**
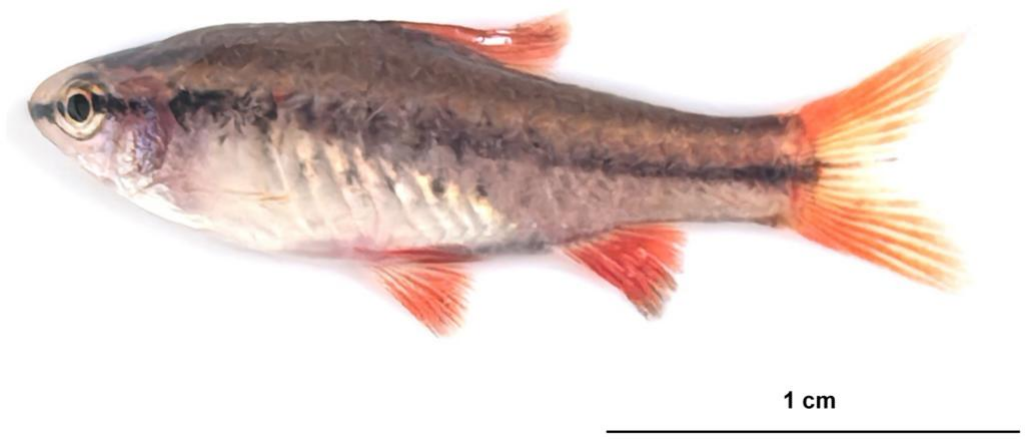
Reference image of *Rohanella titteya*. This photograph was taken by the author of this article, Yu-Hui Tao.

In April 2024, live specimens of *R. titteya* were collected from the Nilwala River, Matara District, Southern Province, Sri Lanka (5°57′00.00 ″N, 80°31′58.80 ″E) and identified to the species level using taxonomic keys as described by Mieno and Karino (2017) and Sudasinghe et al. (2023). Specimens were examined for key traits, including an incomplete lateral line with only 2–5 pored scales. The post-epiphyseal cranial fontanel was absent, gill rakers were reduced (3–7 in number), and the caudal fin had 16 branched rays. Adult males exhibited vivid red body coloration with iridescent green highlights and a faint mid-lateral black stripe. Following collection, the specimens were photographed with a Nikon D850 camera and euthanized using an overdose of eugenol. Post-euthanasia, muscle samples were dissected from the photographed individuals and preserved in 100% ethanol. The euthanized specimens were also preserved in 100% ethanol and subsequently deposited in the zoological specimen room of the College of Ecology at Lishui University. These specimens are cataloged under the voucher number LSU-ZJ2024-04-01 (Figure 1), with Jie Chen (jchen@lsu.edu.cn) serving as the contact person.

Total genomic DNA was extracted from muscle tissues using a Rapid Animal Genomic DNA Isolation Kit (Sangon, Shanghai, China). DNA libraries with a 350-bp insert size were constructed using the TruSeq NanoTM kit (Illumina, San Diego, CA) and sequenced on the Illumina HiSeq 2500 platform, generating 150-bp paired-end reads. Approximately 13.18 Gb of raw data were obtained, with 12.90 Gb retained as clean data after filtering low-quality reads and adapters using Fastp v0.20.0 (Chen et al. 2018). After quality trimming with fastp v0.20.0, reads were mapped to the reference mitogenome of *P. titteya* (GenBank AP011448) with BWA-MEM v0.7.17 (Li 2013) and the mapped subset was assembled with SPAdes v4.10 (Prjibelski et al. 2020), yielding a circular draft contig. To rule out reference bias, the same quality-filtered read set was assembled *de novo* with GetOrganelle v1.7.7 (Jin et al. 2020) (k-mers 21-127); the resulting contig was identical to the SPAdes draft and contained all 37 canonical mitochondrial genes plus two non-coding regions. This 16,715 bp contig was retained as the final mitogenome, circularized and trimmed with MitoZ v2.4 (Meng et al. 2019), and polished twice with Pilon v1.24 (Walker et al. 2014). Genome annotation was performed locally with the stand-alone MITOS2 package (Donath et al. 2019) and cross-validated with NCBI BLAST+ v2.28 (Camacho et al. 2009), GeneWise (Birney et al. 2004), MiTFi (Jühling et al. 2012) and Infernal v1.1 (Nawrocki and Eddy 2013). The tandem repeats was detected using Tandem Repeats Finder (Benson 1999). Subsequently, manual curation was conducted in Geneious Prime v.2024.0.7 (Geneious 2025). A circular genome map was generated with Proksee (Grant et al. 2023). Coverage was evaluated by realigning quality-filtered reads to the mitochondrial assembly with Bowtie2 v2.3.4 (Langmead and Salzberg 2012) and processing the alignments in SAMtools v1.16.1 (Li et al. 2009); per-base depth was then extracted with samtools depth-aa and visualized in R using ggplot2 (Wickham 2016) (Figures S1 and S2).

**Figure 2.**
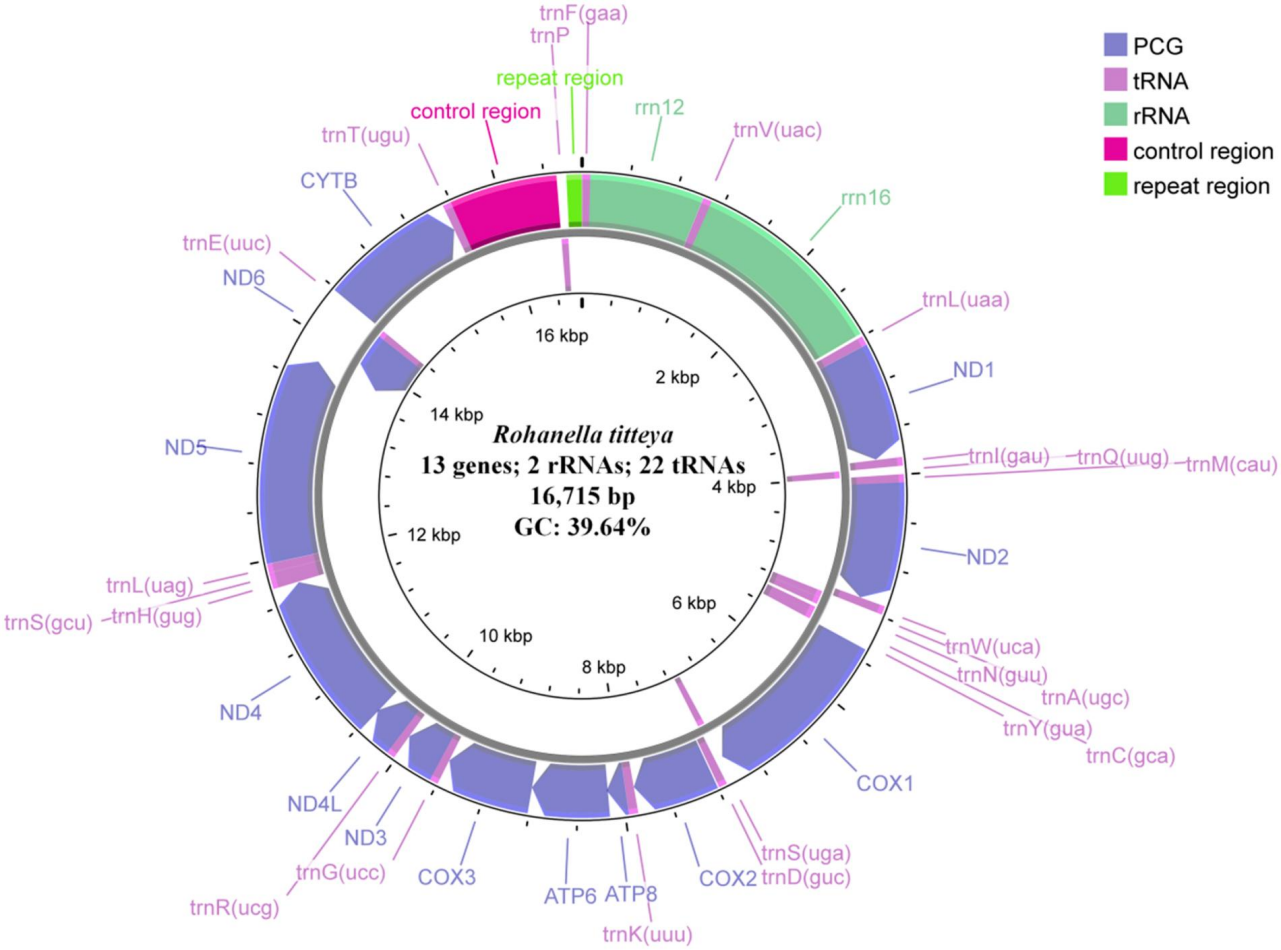
Circular map of the *Rohanella titteya* mitochondrial genome. Arrows indicate transcriptional directions. The control region (D-loop; 904 bp, 32.63% G + C content) is annotated between *trnT* and *trnP* genes.

For our phylogenetic analysis, we used complete mitogenomes currently available for the four genera relevant to the placement of *Rohanella titteya*—namely five species of *Puntius*, three of *Pethia*, two of *Osteochilus*, and one of *Rohanella* (including the new sequence generated here). The well-annotated mitogenome of *Danio rerio* (subfamily Danioninae) served as the out-group (Table 1). The 13 PCG sequences were initially processed through PhyloSuite v1.2.1 (Zhang et al. 2020) for sequence extraction, followed by multiple sequence alignment performed in MAFFT v7.388 (Katoh and Standley 2013). The resulting alignments were merged into a combined matrix and subjected to Bayesian phylogenetic reconstruction using MrBayes 3.2.7 (Ronquist and Huelsenbeck 2003). Model selection analysis implemented in MrModelTest 2.3 (Nylander 2004) determined GTR+F + I as the best-fit nucleotide substitution model. Bayesian inference involved four independent Markov chain Monte Carlo (MCMC) simulations, each iterated over 1 million generations with tree sampling at 1000-generation intervals. A burn-in period eliminating the first 25% of sampled trees (1000 trees) was applied before consensus tree construction. Convergence was confirmed with three complementary diagnostics. Gelman-Rubin PSRFs for every parameter were < 1.01, indicating chain homogeneity. Effective sample sizes exceeded 200 in Tracer v1.7.2 (Rambaut et al. 2018), demonstrating adequate sampling. Trace plots, inspected after discarding the first 25% as burn-in, showed clear stationarity. Independent runs yielded indistinguishable topologies in CompareToBEAST (Chatzou et al. 2018), with branch-length differences < 0.01 substitutions/site and posterior-support variation < 1%. These checks collectively validate the robustness of the Bayesian phylogeny.

**Table 1.** List of species and GenBank accession numbers for the mitogenomes used for the phylogenetic analysis.

Species	GenBank accession	References
*Rohanella titteya*	PQ720779	This study
*Rohanella titteya*	AP011448	Unpublished
*Puntius eugrammus*	AP011369	Unpublished
*Puntius sahyadriensis*	AP012065	Unpublished
*Puntius snyderi*	KC113210	Jang-Liaw et al. (2013)
*Puntius sachsii*	MZ364158	Unpublished
*Puntius paucimaculatus*	OR264466	Unpublished
*Osteochilus salsburyi*	JX220892	Su et al. (2013)
*Osteochilus pentalineatus*	AP011391	Unpublished
*Pethia padamya*	ON864408	Pan et al. (2023)
*Pethia stoliczkana*	OP785085	Qiao et al. (2024)
*Pethia conchonius*	PP059109	Unpublished
*Danio rerio*	AC024175	Broughton et al. (2001)

## Results

The complete mitogenome of *R. titteya* is 16,715 bp long and comprises 13 PCGs, 22 tRNA genes, 2 rRNA genes, a control region (D-loop), and a repeat region. Its nucleotide composition is 32.86% A, 27.50% T, 15.46% G, 24.18% C, giving a G + C content of 39.64%. The majority strand encodes 28 genes (12 PCGs, 14 tRNA genes, and two rRNA genes), whereas the minority strand encodes nine genes (one PCG and eight tRNA genes). All PCGs start with the standard ATN codon and terminate with the stop codon TAA, except *cox2*, *cox3*, *nad2*, *nad3*, *nd4*, and *cytb*, in which a single T codon serves as an incomplete stop codon. The 22 tRNA genes range from 67 bp to 77 bp in length. The *rrn16* and *rrn12* genes are 1,671 bp and 957 bp long, respectively, with G + C contents of 41.05% and 46.71%. The control region (D-loop) is 904 bp long, has a G + C content of 32.63%, and lies between the *trnT* and *trnP* genes (Figure 2). A tandem repeat array (TATATATATATCATAAATTA × 6.2) occurs in the repeat region (positions 16,586-16,712). This region exhibits high read-depth variability (Figures S1 and S2).

**Figure 3.**
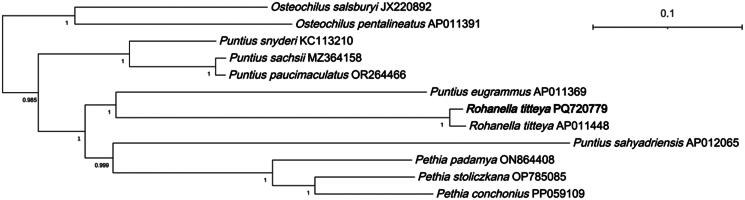
Phylogenetic analysis based on the Bayesian inference method of 13 mitogenome sequences, including the newly sequenced *Rohanella titteya* using 13 protein-coding genes. Numbers at the nodes represent Bayesian’s posterior probabilities. It is rooted with the out-group *Danio rerio* mitogenome sequence (branch not displayed for clarity). The mitogenome sequenced in the present study is highlighted in bold. GenBank accession numbers for the mitogenomic sequences of all species are shown in Table 1. Scale bar indicates substitutions per site.

Phylogenetic reconstruction reveals robust nodal support across key branches. Mitochondrial genome-based topology positions *R. titteya* as the sister taxon to *P. eugrammus* with high confidence. Comparative phylogenetic assessment indicates a mitogenomic affinity to *Puntius*; nuclear evidence is needed before any taxonomic change (Figure 3).

## Discussion and conclusion

This study reports the first complete mitochondrial genome of the *R. titteya* and, on a tree built solely from mitochondrial sequences, places it in the same clade as typical *Puntius* species. Because a mitogenome is a single haplotypic, maternally inherited marker subject to strong selection, its phylogenetic signal often diverges from nuclear-gene or morphological patterns (Funk and Omland 2003; Ballard and Whitlock 2004; Avise 2009). All conclusions here therefore reflect mitochondrial lineage history only and must not be equated directly with the species’ evolutionary history.

When compared with the genus *Rohanella* erected by Sudasinghe et al. 2023 using combined nuclear–mitochondrial markers and morphology, our finding does not create a real “conflict.” Two scenarios plausibly explain the discrepancy: *R. titteya* may still carry an ancestral haplotype shared with *Puntius*, or historical mitochondrial introgression has transferred a *Puntius*-like genome into *R. titteya*, producing mito-nuclear discordance (Funk and Omland 2003; Toews and Brelsford 2012). A single newly sequenced mitogenome is insufficient to settle the systematic position of the genus. Robust resolution will require high-throughput nuclear genomic data with explicit tests for introgression, denser sampling of South-Asian barbs, and the integration of additional morphological characters.

Within this framework our study delivers a validated, gap-free mitogenome for *R. titteya*, providing a molecular tool for rapid population monitoring and conservation; highlights its close mitochondrial affinity to *Puntius*, cautioning against generic assignments based on mitochondria alone; and re-emphasizes the need for cautious interpretation of single-locus evidence, offering a reference case for similar studies. *Rohanella* should for now be treated as a provisional genus, while acknowledging that its type species shows a strong mitochondrial affinity with *Puntius.* Definitive clarification of the boundary between the two genera awaits an integrated analysis of nuclear genomes and morphology.

## Supplementary Material

Figure S1.doc

## Data Availability

The genome sequence data supporting this study are openly available in GenBank of NCBI at https://www.ncbi.nlm.nih.gov under the accession number PQ720779.1. The associated BioProject, SRA, and Biosample numbers are PRJNA1197591, SRR31702435, and SAMN45811546, respectively.

## References

[CIT0001] Avise JC. 2009. Phylogeography: retrospect and prospect. J Biogeogr. 36(1):3–15. doi:10.1111/j.1365-2699.2008.02032.x.

[CIT0002] Ballard JW, Whitlock MC. 2004. The incomplete natural history of mitochondria. Mol Ecol. 13(4):729–744. doi:10.1046/j.1365-294x.2003.02063.x. 15012752

[CIT0003] Benson G. 1999. Tandem repeats finder: a program to analyze DNA sequences. Nucleic Acids Res. 27(2):573–580. doi:10.1093/nar/27.2.573. 9862982 PMC148217

[CIT0004] Broughton RE, Milam JE, Roe BA. 2001. The complete sequence of the zebrafish (*Danio rerio*) mitochondrial genome and evolutionary patterns in vertebrate mitochondrial DNA. Genome Res. 11(11):1958–1967. doi:10.1101/gr.156801. 11691861 PMC311132

[CIT0005] Birney E, Clamp M, Durbin R. 2004. genewise and genomewise. Genome Res. 14(5):988–995. doi:10.1101/gr.1865504. 15123596 PMC479130

[CIT0006] Camacho C, Coulouris G, Avagyan V, Ma N, Papadopoulos J, Bealer K, Madden TL. 2009. BLAST+: architecture and applications. BMC Bioinformatics. 10(1):421. doi:10.1186/1471-2105-10-421. 20003500 PMC2803857

[CIT0007] Chatzou M, Floden EW, Di Tommaso P, Gascuel O, Notredame C. 2018. Generalized bootstrap supports for phylogenetic analyses of protein sequences incorporating alignment uncertainty. Syst Biol. 67(6):997–1009. doi:10.1093/sysbio/syx096. 30295908

[CIT0008] Chen SF, Zhou YQ, Chen YR, Gu J. 2018. Fastp: an ultra-fast all-in-one FASTQ preprocessor. Bioinformatics. 34(17):i884–i890. doi:10.1093/bioinformatics/bty560. 30423086 PMC6129281

[CIT0009] Donath A, Jühling F, Al-Arab M, Bernhart SH, Reinhardt F, Stadler PF, Middendorf M, Bernt M. 2019. Improved annotation of protein-coding genes boundaries in metazoan mitochondrial genomes. Nucleic Acids Res. 47(20):10543–10552. doi:10.1093/nar/gkz833. 31584075 PMC6847864

[CIT0010] Funk DJ, Omland KE. 2003. Species-level paraphyly and polyphyly: frequency, causes and consequences. Annu Rev Ecol Evol Syst. 34(1):397–423. doi:10.1146/annurev.ecolsys.34.011802.132421.

[CIT0011] Geneious. 2025. https://www.geneious.com.

[CIT0012] Grant JR, Enns E, Marinier E, Mandal A, Herman EK, Chen C-Y, Graham M, Van Domselaar G, Stothard P. 2023. Proksee: in-depth characterization and visualization of bacterial genomes. Nucleic Acids Res. 51(W1):W484–W492. doi:10.1093/nar/gkad326. 37140037 PMC10320063

[CIT0013] Jang-Liaw NH, Chang CH, Tsai CL. 2013. Complete mitogenomes of two *Puntius* in Taiwan: *P. semifasciolatus* and *P. snyderi* (Cypriniformes: Cyprinidae). Mitochondrial DNA. 24(3):228–230. doi:10.3109/19401736.2012.752472. 23327461

[CIT0014] Jin JJ, Yu WB, Yang JB, Song Y, DePamphilis CW, Yi TS, Li DZ. 2020. GetOrganelle: a fast and versatile toolkit for accurate *de novo* assembly of organelle genomes. Genome Biol. 21(1):241. doi:10.1186/s13059-020-02154-5. 32912315 PMC7488116

[CIT0015] Jühling F, Pütz J, Bernt M, Donath A, Middendorf M, Florentz C, Stadler PF. 2012. Improved systematic tRNA gene annotation allows new insights into the evolution of mitochondrial tRNA structures and into the mechanisms of mitochondrial genome rearrangements. Nucleic Acids Res. 40(7):2833–2845. doi:10.1093/nar/gkr1131. 22139921 PMC3326299

[CIT0016] Katoh K, Standley DM. 2013. MAFFT multiple sequence alignment software version 7: improvements in performance and usability. Mol Biol Evol. 30(4):772–780. doi:10.1093/molbev/mst010. 23329690 PMC3603318

[CIT0017] Langmead B, Salzberg SL. 2012. Fast gapped-read alignment with Bowtie 2. Nat Methods. 9(4):357–359. doi:10.1038/nmeth.1923. 22388286 PMC3322381

[CIT0018] Li H. 2013. Aligning sequence reads, clone sequences and assembly contigs with BWA-MEM. arXiv Preprint arXiv:1303.3997.

[CIT0019] Li H, Handsaker B, Wysoker A, Fennell T, Ruan J, Homer N, Marth G, Abecasis G, Durbin R, 1000 Genome Project Data Processing Subgroup. 2009. The sequence alignment/map format and SAMtools. Bioinformatics. 25(16):2078–2079. doi:10.1093/bioinformatics/btp352. 19505943 PMC2723002

[CIT0020] Mieno A, Karino K. 2017. Sexual dimorphism and dichromatism in the cyprinid fish *Puntius titteya*. Ichthyol Res. 64(2):250–255. doi:10.1007/s10228-016-0559-y.

[CIT0021] Meng GL, Li YY, Yang CT, Liu SL. 2019. MitoZ: a toolkit for animal mitochondrial genome assembly, annotation and visualization. Nucleic Acids Res. 47(11):e63–e63-e63–e63. doi:10.1093/nar/gkz173. 30864657 PMC6582343

[CIT0022] Nawrocki EP, Eddy SR. 2013. Infernal 1.1: 100-fold faster RNA homology searches. Bioinformatics. 29(22):2933–2935. doi:10.1093/bioinformatics/btt509. 24008419 PMC3810854

[CIT0023] Nylander JAA. 2004. MrModeltest v2 program distributed by the author. Uppsala: Evolutionary Biology Centre, Uppsala University.

[CIT0024] Palmer-Newton A, de Alwis Goonatilake S, Fernado M, Kotagama O. 2019. *Puntius titteya* (errata version published in 2020). The IUCN Red List of Threatened Species. e.T18900A174843175.

[CIT0025] Pan Y, Xiang X, Tong Y, Hu S, Zhou D, Wu G, Qin Y. 2023. The complete mitochondrial genome of *Pethia padamya* (Actinopteri, Cyprinidae). Mitochondrial DNA B Resour. 8(3):426–429. doi:10.1080/23802359.2023.2186724. 36998786 PMC10044152

[CIT0026] Prjibelski A, Antipov D, Meleshko D, Lapidus A, Korobeynikov A. 2020. Using SPAdes de novo assembler. Curr Protoc Bioinformatics. 70(1):e102. doi:10.1002/cpbi.102. 32559359

[CIT0027] Qiao Z, Xing J, Li F. 2024. Structural analysis and phylogenetic relationships of a teleost fish, *pethia stoliczkana* based on the complete mitochondrial genome sequence. PJZ. 56(2):853. doi:10.17582/journal.pjz/20230302100304.

[CIT0028] Rambaut A, Drummond AJ, Xie D, Baele G, Suchard MA. 2018. Posterior summarisation in Bayesian phylogenetics using Tracer 1.7. Syst Biol. 67(5):901–904. doi:10.1093/sysbio/syy032. 29718447 PMC6101584

[CIT0029] Ronquist F, Huelsenbeck JP. 2003. MrBayes 3: Bayesian phylogenetic inference under mixed models. Bioinformatics. 19(12):1572–1574. doi:10.1093/bioinformatics/btg180. 12912839

[CIT0030] Su LW, Liu ZZ, Tang WQ, Liu D, Wu CY, Yang JQ. 2013. Complete mitochondrial genome of *Osteochilus salsburyi* (Cypriniformes, Cyprinidae). Mitochondrial DNA. 24(3):252–254. doi:10.3109/19401736.2012.752482. 23324059

[CIT0031] Sudasinghe H, Rüber L, Meegaskumbura M. 2023. Molecular phylogeny and systematics of the South Asian freshwater‐fish genus *Puntius* (Teleostei: Cyprinidae). Zool Scr. 52(6):571–587. doi:10.1111/zsc.12618.

[CIT0032] Sundarabarathy TV, Edirisinghe U, Dematawewa CMB. 2004. Captive breeding and rearing of fry and juveniles of Cherry barb (*Puntius titteya* Deraniyagala), a highly threatened endemic fish species in Sri Lanka. Tropical Agricultural Research. 16:137–149.

[CIT0033] Toews DPL, Brelsford A. 2012. The biogeography of mitochondrial and nuclear discordance in animals. Mol Ecol. 21(16):3907–3930. doi:10.1111/j.1365-294X.2012.05664.x. 22738314

[CIT0034] Walker BJ, Abeel T, Shea T, Priest M, Abouelliel A, Sakthikumar S, Cuomo CA, Zeng Q, Wortman J, Young SK, et al. 2014. Pilon: an integrated tool for comprehensive microbial variant detection and genome assembly improvement. PLoS One. 9(11):e112963. doi:10.1371/journal.pone.0112963. 25409509 PMC4237348

[CIT0035] Wickham H. 2016. Data Analysis. In: Wickham H, editor. Ggplot2: elegant graphics for data analysis. Cham: springer International Publishing, p. 189–201.

[CIT0036] Zhang D, Gao F, Jakovlić I, Zou H, Zhang J, Li WX, Wang GT. 2020. PhyloSuite: an integrated and scalable desktop platform for streamlined molecular sequence data management and evolutionary phylogenetics studies. Mol Ecol Resour. 20(1):348–355. doi:10.1111/1755-0998.13096. 31599058

