# Supplementary material for: The first complete mitochondrial genome of Rohanella titteya (Cypriniformes: Cyprinidae) and its phylogenetic analysis
Source: Mitochondrial DNA B Resour. 2025 Jun 18;10(7):590–4. doi: 10.1080/23802359.2025.2519215 (PMC12180331; doi:10.1080/23802359.2025.2519215)
Supplement: Figure S1.doc [file TMDN_A_2519215_SM5715.doc]

**The first complete mitochondrial genome of *Rohanella titteya* (Cypriniformes: Cyprinidae) and its phylogenetic analysis**


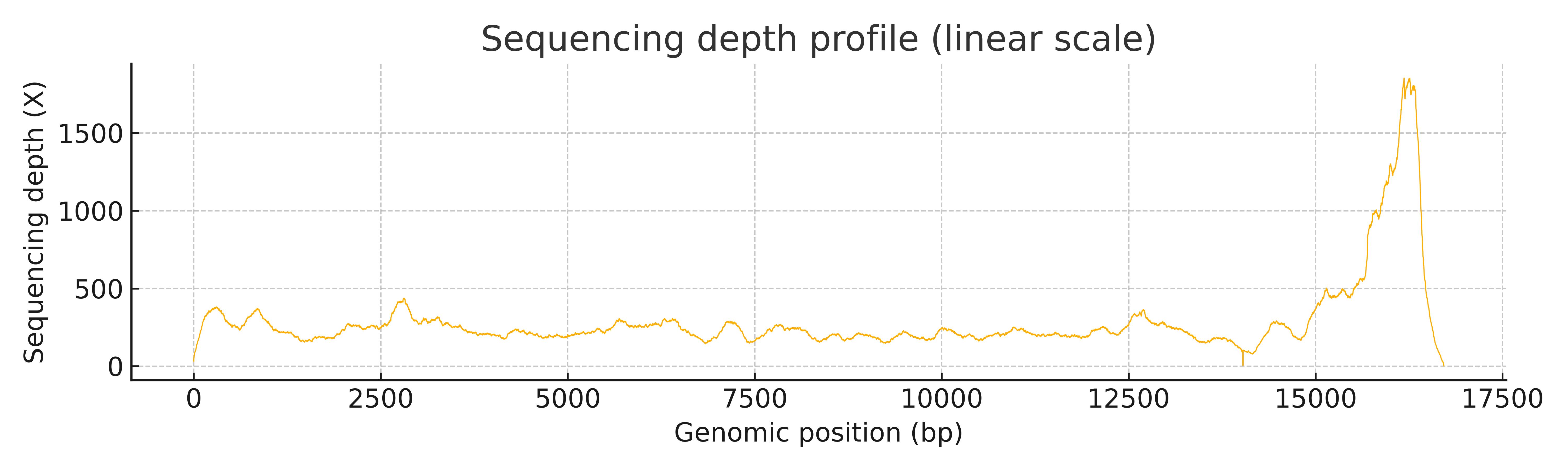


**Figure S1.** Linear sequencing-depth profile of the *Rohanella titteya* mitochondrial genome. X-axis: genomic position (bp) along the mitochondrial genome of *R. titteya*. Y-axis: per-base sequencing depth plotted on a linear scale (×).


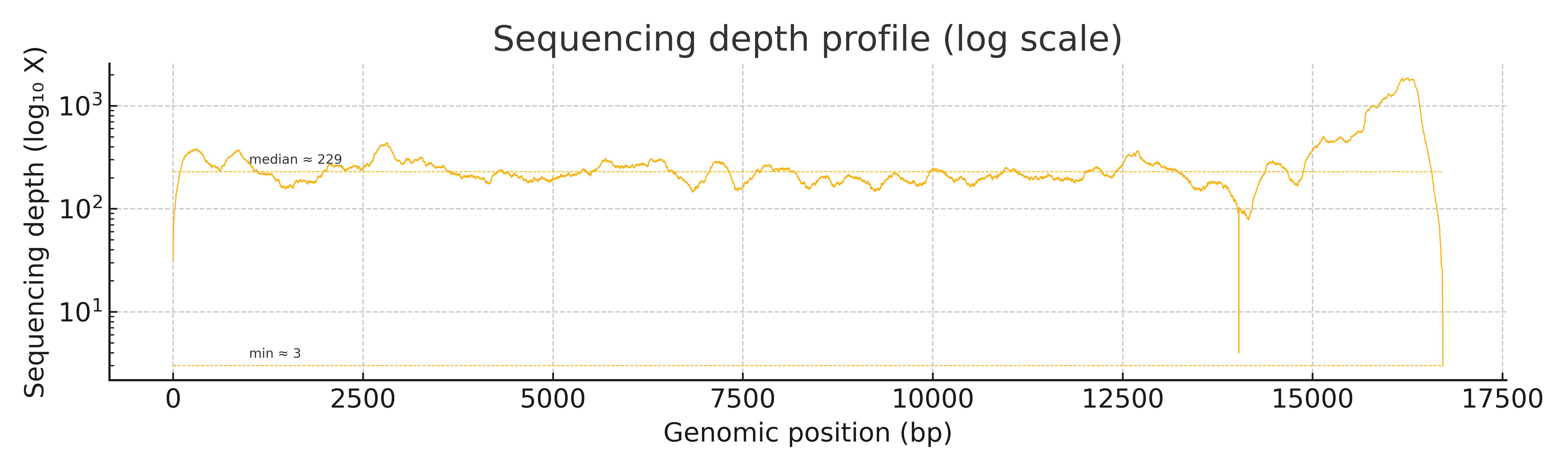


**Figure S2.** Log-scaled per-base sequencing depth across the mitochondrial genome of *Rohanella titteya*. X-axis: genomic position (bp) along the mitochondrial genome of *R. titteya*. Y-axis: per-base sequencing depth plotted on a log₁₀scale (×).
